# Cognitive reserve hypothesis in frontotemporal dementia: A FDG-PET study

**DOI:** 10.1016/j.nicl.2020.102535

**Published:** 2020-12-16

**Authors:** Leonie Beyer, Johanna Meyer-Wilmes, Sonja Schönecker, Jonas Schnabel, Julia Sauerbeck, Maximilian Scheifele, Catharina Prix, Marcus Unterrainer, Cihan Catak, Oliver Pogarell, Carla Palleis, Robert Perneczky, Adrian Danek, Katharina Buerger, Peter Bartenstein, Johannes Levin, Axel Rominger, Michael Ewers, Matthias Brendel

**Affiliations:** a Dept. of Nuclear Medicine, University Hospital, Ludwig-Maximilians-Universität München, Marchioninistr. 15, 81377 Munich, Germany; b Dept. of Neurology, University Hospital, Ludwig-Maximilians-Universität München, Marchioninistr. 15, 81377 Munich, Germany; c Department of Radiology, University Hospital, Ludwig-Maximilians-Universität München, Marchioninistr. 15, 81377 Munich, Germany; d Institute for Stroke and Dementia Research, University Hospital, Ludwig-Maximilians-Universität München, Feodor-Lynen-Str. 17, 81377 Munich, Germany; e Dept. of Psychiatry, University Hospital, Ludwig-Maximilians-Universität München, Nußbaumstr. 7, 80336 Munich, Germany; f DZNE – German Center for Neurodegenerative Diseases, Feodor-Lynen-Str. 17, 81377 Munich, Germany; g Munich Cluster for Systems Neurology (SyNergy), Feodor-Lynen-Str. 17, 81377 Munich, Germany; h Dept. of Nuclear Medicine, University of Bern, Inselspital, Freiburgstr. 18, 3010 Bern, Switzerland; i Ageing Epidemiology (AGE) Research Unit, School of Public Health, Imperial College, Level 2, Faculty Building, South Kensington Campus, London SW7 2AZ, United Kingdom

**Keywords:** Cognitive reserve, Frontotemporal dementia, FDG-PET, Hypometabolism

## Abstract

**Background and objective:**

Reserve is defined as the ability to maintain cognitive functions relatively well at a given level of pathology. Early life experiences such as education are associated with lower dementia risk in general. However, whether more years of education guards against the impact of brain alterations also in frontotemporal dementia (FTD) has not been shown in a large patient collective. Therefore, we assessed whether education is associated with relatively high cognitive performance despite the presence of [^18^F]-fluorodeoxyglucose positron-emission-tomography (FDG-PET) hypometabolism in FTD.

**Methods:**

Sixty-six FTD subjects (age 67 ± 8 years) and twenty-four cognitively healthy controls (HC) were evaluated. Brain regions with FTD-related glucose hypometabolism in the contrast against HC and brain regions that correlate with the cognitive function were defined by a voxel-based analysis and individual FDG-PET values were extracted from all frontotemporal brain areas. Linear regression analysis served to test if education is associated with residualized cognitive performance and regional FDG-PET hypometabolism after controlling for global cognition.

**Results:**

Compared to healthy controls, patients with FTD showed glucose hypometabolism in bilateral frontal and temporal brain areas whereas cognition was only associated with deteriorated glucose metabolism in the left temporal lobe. The education level was significantly correlated with the residualized cognitive performance (residuals from regression analysis between hypometabolism and cognitive function as a quantitative index of reserve) and also negatively correlated with left temporal FDG-PET hypometabolism after controlling for cognition.

**Conclusions:**

In patients with FTD, the education level predicts the existing left temporal FDG-PET hypometabolism at the same cognition level, supporting the cognitive reserve hypothesis in FTD.

## Highlights

• The cognitive reserve hypothesis is also applicable for FTD.
• The educational level predicts left-temporal hypometabolism in FTD.
• Residualized cognitive performance is positively correlated with education.
• Residualized cognitive performance is negatively correlated with hypometabolism.

## 1 Introduction

Among the spectrum of neurodegenerative diseases, frontotemporal dementia (FTD) is the second most common cause of presenile early onset dementia (Vieira et al., 2013). The prevalence of FTD ranges from 1 to 22 per 100 000 (Lambert et al., 2014, Knopman and Roberts, 2011). According to clinical criteria, the core forms of FTD include behavioural-variant FTD (bvFTD) (Rascovsky et al., 2011), non-fluent variant primary progressive aphasia (nfPPA) and semantic-variant PPA (svPPA) (Bang et al., 2015). In subjects with FTD, positron emission tomography (PET) with [^18^F]-fluorodeoxyglucose (FDG) allows to detect a characteristic pattern of FDG-PET hypometabolism in frontal and temporal lobe regions (Ishii et al., 1998, Grimmer et al., 2004) which distinguishes FTD from other neurodegenerative diseases such as Alzheimer’s disease (AD) (Foster et al., 2007). Although FDG-PET hypometabolism is predictive of lower cognitive performance in FTD (Nazem et al., 2018, Robb et al., 2017, Machulda et al., 2017), protective environmental factors such as education may modulate the association between pathological brain alterations and cognitive impairment. The theory of cognitive reserve postulates that life experiences that are cognitively stimulating, such as education, may enhance the capability to maintain cognitive performance relatively well in aging and disease (Stern et al., 1992, STERN, 2002). Support for the hypothesis of protective effects of education has been best established in AD so far (Stern, 2012). At preclinical, prodromal or dementia stages of AD, more years of education were associated with stronger FDG-PET hypometabolism when controlling for cognitive performance (Perneczky et al., 2006, Ewers et al., 2013), suggesting that patients with higher education can sustain similar levels of cognitive performance in the presence of more severe pathology. However, in FTD, only few studies have investigated the association of proxy measures of reserve and cognition. Higher education was negatively associated with FDG-PET and brain perfusion in the bilateral frontal cortex for FTD subjects when controlled for demographic variables and cognitive performance (Perneczky et al., 2007, Borroni et al., 2009, Maiovis et al., 2018) whereas a similar association was observed in the left inferior temporal, parahippocampal, and supramarginal gyri in a small cohort of eleven subjects with nfPPA (Perneczky et al., 2007b). In summary, these studies provided indirect evidence that higher education allows to tolerate more pathology at a given level of cognition. However, they did not quantify reserve, i.e. the extent to which cognition was higher than expected based on a given level of pathology. Here we used the residualization approach to derive a quantitative index of reserve. In regression analytical analysis, the residual of cognitive performance after accounting for pathological brain alterations were derived, where higher positive residuals indicate higher cognitive performance than expected based on FTD brain pathology. As the pathological marker we used regional FDG-PET hypometabolism, which is predictive of global cognition. Our primary hypothesis was that more years of education is positively associated with cognitive residuals, i.e. indicating that higher education is associated with higher resilience of cognitive performance relative to a given level of FDG-PET hypometabolism in clinically confirmed FTD.

## 2 Material and methods

### 2.1 Study design and patient enrollment

The study entailed patients with a clinical diagnosed FTD at the time of PET imaging (Rascovsky et al., 2011, Gorno-Tempini et al., 2011). Suspected tau positive FTD cases presenting with progressive supranuclear palsy or corticobasal syndrome phenotype (Höglinger et al., 2017) were not included in the analysis. The clinical phenotype was nfPPA in eight cases, svPPA in 34 cases, and bvFTD in 24 cases. All subjects were recruited at the University Hospital of Munich, and were scanned in a clinical setting at the Department of Nuclear Medicine between 2010 and 2017. Patients were referred by the Departments of Neurology, Psychiatry and Institute for Stroke and Dementia Research. All subjects underwent clinical dementia workup including detailed cognitive testing and FDG-PET. Inclusion was performed for all levels of cognitive impairment as long as patients met criteria for FTD. All patients maintained a clinical diagnosis of FTD during a mean follow up of 21 months.

### 2.2 Clinical assessment and cognitive testing

A clinical neurological examination and neuropsychological testing including Mini-Mental-State Examination (MMSE) (Folstein et al., 1975) assessment was performed in all FTD patients. Years of education (YoE) were recorded, and laboratory parameters for metabolic causes of cognitive impairment (vitamin B_12_, thiamine and folate levels, thyroid and liver function) were assessed.

### 2.3 FDG-PET acquisition and pre-processing

FDG was purchased commercially. FDG-PET images of both FTD patients and healthy controls were acquired using a 3-dimensional GE Discovery 690 PET/CT scanner or a Siemens ECAT EXACT HR + PET scanner. All patients fasted for at least six hours, and had a plasma glucose level <120 mg/dl (6.7 mM) at time of scanning. A dose of 142 ± 8 MBq FDG was injected as a slow intravenous bolus while the subject sat quietly in a room with dimmed light and low noise level. A static emission frame was acquired from 30 min to 50 min p.i. for the GE Discovery 690 PET/CT, or from 30 to 60 min p.i. for the Siemens ECAT EXACT HR + PET scanner. A low-dose CT scan (GE) or a transmission scan with external ^68^Ge-sources (Siemens) was performed prior to the static acquisition for attenuation correction. PET data were reconstructed iteratively (GE) or with filtered back-projection (Siemens).

All individual FDG-PET image volumes were spatially normalized to an in-house FDG-PET template (voxel-size 2 × 2 × 2 mm) within the MNI space (Daerr et al., 2017) using PMOD software (version 3.5, PMOD Technologies Ltd., Zürich, Switzerland). Non-linear warping and transient input smoothing (8 × 8 × 8 mm) was used for the spatial normalization. An in house database of 24 age matched cognitively normal individuals served as FDG-PET template and was also used for group wise comparisons. Scaling by a reference region containing the whole cerebellum and the brainstem served for normalization to standardized-uptake-value-ratio (SUVr) images and regional values of predefined brain regions of the Hammer’s atlas (Yakushev et al., 2008, Dukart et al.,, Hammers et al., 2003). A Gaussian filter of 8 mm was used prior to voxel-wise comparisons.

### 2.4 Statistical analysis

In order to identify regional FDG-PET hypometabolism in the FTD patients, we performed a voxel-wise comparison between FTD subjects and cognitively healthy controls using an unpaired *t*-test with family-wise error rate (FWE) correction for multiple comparisons (p < 0.05, FWE-corrected, k > 50 voxel) with age and sex as covariates. In sub-analyses, those voxel-wise comparisons were repeated for (i) the bvFTD/ PPA subgroup against HC and (ii) subjects with lower/ higher education levels (≤12 years/>12 years).

In order to identify which areas of FDG-PET alterations were associated with global cognition, i.e. were of relevance for differences in cognitive abilities, we tested in a separate voxel-wise regression analyses the association between MMSE and FDG-PET SUVr, controlling for age and sex (p < 0.05, FWE-corrected, k > 50 voxel). This latter analysis resulted in a single cluster, which overlapped with regions of FDG-PET hypometabolism (in the left temporal cortex). FDG-PET values were averaged across voxels within that cluster and were used for the subsequent residualization approach.

Voxel-based SUVr values from clusters of significant hypometabolism were extracted using PMOD (V3.5, PMOD technologies, Basel, Switzerland) and averaged across voxels within each cluster for further analysis. Quantitative FDG-PET SUVr were compared between phenotypes of FTD and HC, respectively, in the whole frontotemporal and the left temporal cluster using student’s *t*-test.

In order to residualize global cognitive performance as a measure of reserve in the FTD patients, we employed in linear regression analysis MMSE as the dependent variable, and with FDG-PET SUVr cluster value (identified in the previous analysis step, see above) as the main predictor, controlled for age and sex. This resulted in residualized MMSE values as our primary index of reserve. In a secondary analysis, we tested whether we can replicated previous results of a negative association between education and FDG-PET when controlling for global cognitive performance, i.e. we used linear regression analysis with FDG-PET SUVr cluster values as the dependent variable with the education level (YoE) as the predictor controlled for MMSE, age, sex. Regression analyses were performed separately for all single frontal and temporal regions and p-values were controlled for multiple testing with the Benjamini-Hochberg procedure (Benjamini and Hochberg, 1995).

SPSS (version 25, IBM, Chicago, IL) was used for all statistical and Statistical Parametric Mapping (SPM) 12 (Wellcome Department of Cognitive Neurology) implemented in MATLAB (version 2016; MathWorks Inc.) for voxel-wise analyses. A significance level of p < 0.05 was applied in all analyses (with additional FWE correction for all SPM analyses).

## 3 Results

### 3.1 Demographics

The study population consisted of 66 individuals (50% male) with a clinical diagnosis of FTD (n = 24 bvFTD, n = 42 PPA). Mean MMSE was 24.0 (±5.5) at the time of clinical dementia work-up. For details of the study population see table 1.

**Table 1:** Demographics, covariates, education and cognitive testing results of the study population. FTD, frontotemporal dementia; PPA, primary progressive aphasia; bvFTD, behavioural-variant FTD; HC, healthy control; SD, standard deviation; MMSE, mini-mental-state examination; TMT, Trail Making Test.

	FTD	PPA	bvFTD	HC
Number of subjects	66	42	24	24
Age (y, mean ± SD)	66.9 ± 8.3	68.9 ± 7.6	63.4 ± 9.3	64.5 ± 7.3
Sex (♂ / ♀)	♂ 33 / ♀ 33	♂ 18 / ♀ 24	♂ 15 / ♀ 9	♂ 15 / ♀ 9
Education (y, mean ± SD)	12.5 ± 3.0	12.7 ± 3.1	12.1 ± 2.9	
Follow-up (m, mean ± SD)	20.9 ± 19.8	25.5 ± 22.5	14.7 ± 13.7	

Neuropsychological evaluation				
MMSE (mean ± SD)	24.0 ± 5.5	23.2 ± 6.2	25.5 ± 4.0	30 ± 0
TMT-A (mean ± SD)	24.7 ± 24.9	15.3 ± 16.3	34.1 ± 28.9	
TMT-B (mean ± SD)	106.4 ± 77.3	72.2 ± 27.4	137.8 ± 95.0	
Naming (mean ± SD)	8.1 ± 4.6	5.6 ± 3.6	10.8 ± 4.0	
Semantic verbal fluency (mean ± SD)	8.7 ± 4.4	6.6 ± 3.5	11.0 ± 4.3	
Phonetic verbal fluency (mean ± SD)	9.1 ± 4.7	8.2 ± 2.7	10.0 ± 6.0	

### 3.2 Regional neuronal injury patterns and cognition

**Fig. 1:**
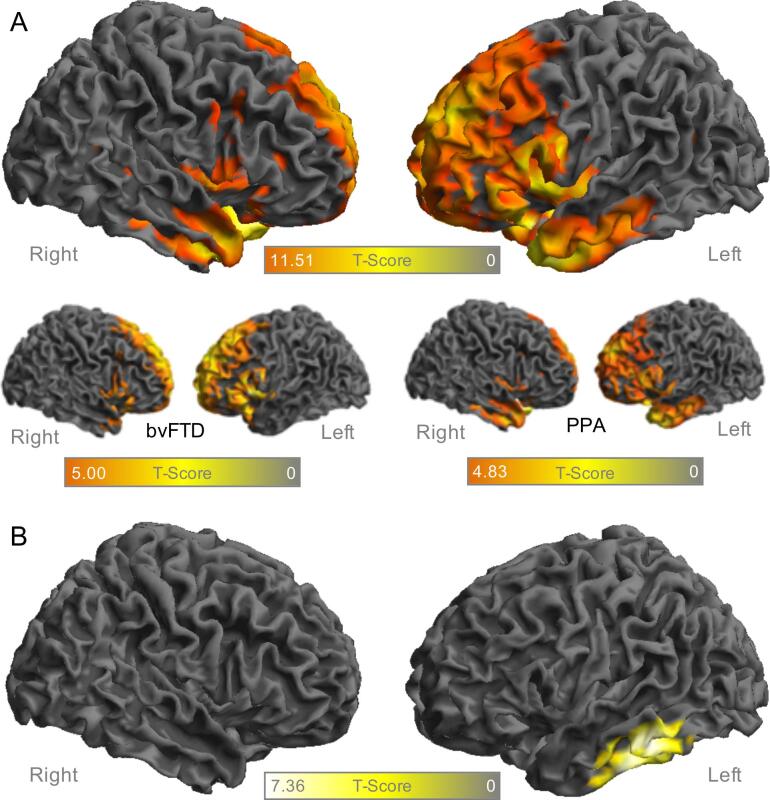
Surface projections of the right (on the left) and left hemisphere (on the right) in the FTD cohort (n = 66) **A** Regions with significant hypometabolism (p < 0.05, FWE-corrected, k > 50 voxel) in FDG-PET against healthy controls (n = 24) for the whole cohort and separately for bvFTD and PPA **B** Correlation cluster of FDG-PET with MMSE (p < 0.05, FWE-corrected, k > 50 voxel) for the whole FTD cohort. FDG, fluordesoxyglucose; FTD, frontotemporal dementia; FWE, family-wise error rate; PPA, primary progressive aphasia; bvFTD, behavioural-variant FTD; MMSE, mini-mental-state-examination; PET, positron-emission-tomography.

Compared to healthy controls, patients with FTD showed lower FDG-PET in frontal and temporal cortices in voxel-wise analysis (Fig. 1A), using p values of <0.05 controlled for multiple comparisons by FWE correction. The analysis revealed one large bilateral fronto-temporal cluster with a peak at −34/8/−26 mm (67878 voxels, T-Score 11.51) and two sub-peaks at 0/8/−18 mm (T-Score 10.75) and −26/4/−26 mm (T-Score 10.49). Compared to healthy controls, the majority of patients showed a significantly reduced glucose metabolism in the frontotemporal cluster (SUVr < 2 standard deviations from the mean of HC) for both phenotypes (PPA: 27/42, bvFTD: 14/24). The hypometabolism in the frontotemporal cluster was not significantly different between PPA and bvFTD phenotypes (SUVr 0.757 vs 0.771, p = 0.208). The hypometabolism pattern of patients with bvFTD and PPA phenotype was similar to the whole FTD cohort, but the PPA subgroup indicated the expected emphasis in temporal cortices, whereas there was a stronger involvement of frontal cortices in bvFTD.

In order to test which FDG-PET alterations are associated with a decrease in cognitive performance, we computed voxel wise analysis with MMSE as the predictor, controlled for age and sex. Results showed that lower MMSE was associated with lower FDG-PET in a solitary cluster within the lateral left-temporal lobe (Fig. 1B) with a peak at −60/–22/−34 (1814 voxels, T-Score 7.36), which overlapped with the hypometabolic regions.

Comparing the FDG-PET values in this cluster between HC and FTD phenotypes, both PPA and bvFTD patients showed significantly reduced glucose metabolism (PPA: p < 0.001, bvFTD: p = 0.025).

We used the averaged FDG-PET uptake in this left temporal cluster for the subsequent analysis on reserve, i.e. global cognition that is higher than expected based on FDG-PET hypometabolism.

To validate the left temporal cluster extracted in the voxel-wise analysis, we performed atlas based regional correlations of all brain areas with a significant hypometabolism in FTD patients. Again, only FDG-PET SUVr in left temporal brain regions indicated a significant association with MMSE after controlling for age, sex and multiple comparisons (see Table 2).

**Table 2:** Regression coefficients of the correlation of glucose metabolism in FDG-PET and cognitive performance for all frontal and temporal cortical regions (covariate age and sex). β, regression coefficient; ***p-value < 0.001, **p value < 0.01, *p-value < 0.05. P-values are reported after false-discovery-rate correction for multiple comparisons. Age and sex served as covariates.

Cortical region	Left Hemisphere		Right Hemisphere	
	β	p-value	β	p-value
Frontal lobe - mid frontal gyrus	−0.045	0.831	−0.144	0.463
Frontal lobe - straight gyrus	0.309	0.072	0.202	0.320
Frontal lobe - orbitofrontal cortex - anterior orbital gyrus	0.157	0.498	0.015	0.947
Frontal lobe - inferior frontal Gyrus	0.061	0.769	−0.110	0.566
Frontal lobe - superior frontal Gyrus	−0.087	0.671	−0.134	0.472
Frontal lobe - orbitofrontal cortex -medial orbital gyrus	0.189	0.344	0.099	0.623
Frontal lobe - orbitofrontal cortex -lateral orbital gyrus	0.085	0.660	−0.032	0.880
Frontal lobe - orbitofrontal cortex -posterior orbital gyrus	0.218	0.278	0.007	0.977
Anterior cingulus - presubgenual cortex	0.333	0.051	0.149	0.482
Subcallosal area	0.240	0.233	0.163	0.472
Anterior cingulus - presubgenual cortex	0.214	0.280	0.111	0.551
Hippocampus	0.219	0.282	0.070	0.732
Amygdala	0.148	0.484	0.127	0.500
Anterior temporal lobe - medial	0.364	0.018*	0.193	0.325
Anterior temporal lobe – inferior lateral	0.464	0.001**	0.152	0.486
Parahippocampal gyrus	0.301	0.073	0.072	0.740
Posterior superior temporal gyrus	0.250	0.260	−0.143	0.486
Middle and inferior temporal gyrus	0.547	<0.001***	0.122	0.512
Fusiform gyrus	0.308	0.069	0.046	0.848
Posterior temporal lobe	0.501	<0.001***	0.005	0.966
Temporal anterior superior gyrus	0.454	0.002**	0.145	0.447
Cingulate gyrus - anterior part	0.019	0.946	−0.038	0.859

### 3.3 Education as a predictor of residualized cognitive performance

**Fig. 2:**
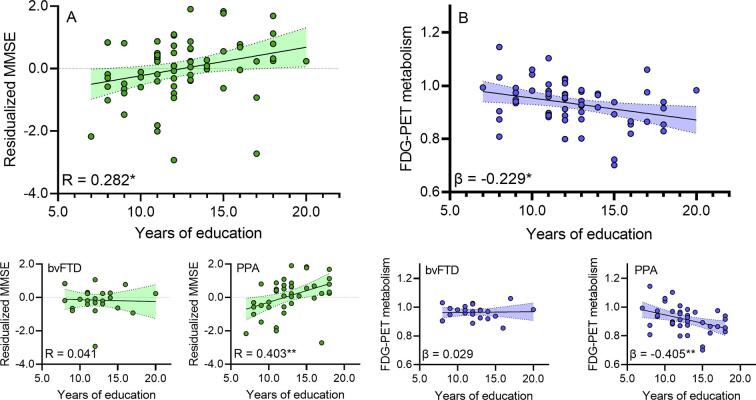
**A** Correlation plots showing the residualized FDG-PET/ MMSE scores as a function of years of education for the whole FTD cohort and separately for the bvFTD and PPA subgroup. **B** Regression plots showing FDG-PET in the left temporal cluster as a function of years of education after control for age, sex and MMSE for the whole FTD cohort and separately for the bvFTD and PPA subgroup.. *p-value < 0.05. FDG, fluordesoxyglucose; MMSE, mini-mental-state-examination; PET, positron-emission-tomography; FTD, frontotemporal dementia; PPA, primary progressive aphasia; bvFTD, behavioural-variant FTD.

We tested whether higher education is associated with higher cognitive residuals after accounting for the influence of FDG-PET hypometabolism present in the previously identified left temporal cluster. Results of the regression analysis showed that more years of education were associated with higher residualized MMSE, i.e. higher MMSE than expected based on the level of left-frontal FDG-PET hypometabolism in the FTD patients (R = 0.282, p = 0.022, see Fig. 2A). In the separate analysis of PPA and bvFTD phenotypes, the correlation was even higher for the isolated PPA cohort (R = 0.403, p = 0.008), but did not reach significance in bvFTD (R = 0.041, p = 0.850).

In a secondary analysis, we tested whether at a given level of cognitive performance, higher education is associated with differences in the FDG-PET metabolism in the inferior temporal lobe cluster. More years of education were associated with lower FDG-PET SUVr (β = −0.229, p = 0.015, see Fig. 2B) when controlling for MMSE, age, sex, suggesting that subjects with higher education can tolerate more FDG-PET hypometabolism. This significant association was reproduced in the PPA subgroup (β = -0.405, p = 0.008), but not in bvFTD (β = 0.029, p = 0.892).

**Fig. 3:**
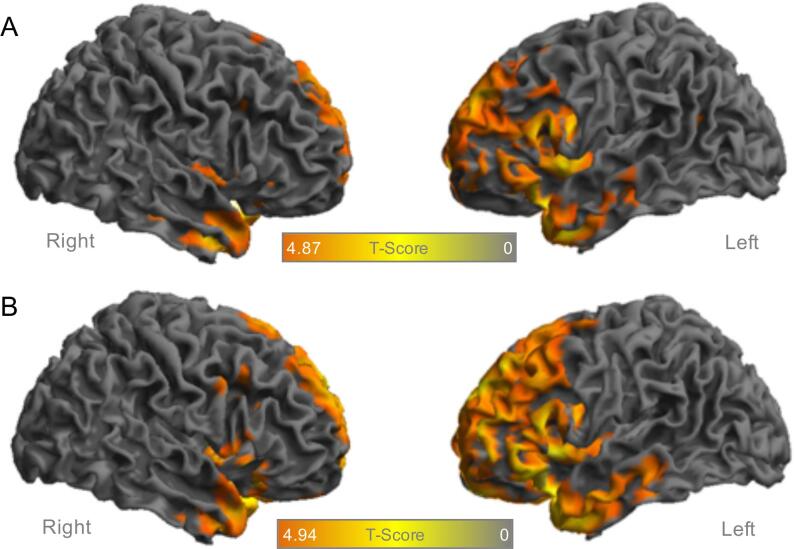
Surface projections of the right (on the left) and left hemisphere (on the right) in the FTD cohort with significant hypometabolism (p < 0.05, FWE-corrected, k > 50 voxel) in FDG-PET against healthy controls (n = 24) separately for subjects with **A** lower education levels (n = 37) and **B** higher education levels (n = 29). FDG, fluordesoxyglucose; FTD, frontotemporal dementia; FWE, family-wise error rate; PET, positron-emission-tomography.

To test if subjects with lower education still have a detectable frontotemporal hypometabolism when compared to HC, we divided the FTD cohort in two subgroups of lower/ higher education level (≤12 years/>12 years). Although the hypometabolism was more pronounced in the subgroup with high education, the voxel-wise comparison still revealed significant clusters of hypometabolism in frontal and temporal cortices in the subgroup with low education (see Fig. 3). Thus, FDG-PET indicated high enough sensitivity for detection of glucose metabolism alterations in patients with FTD and low education.

## 4 Discussion

In the present study, we found higher education to be associated with ameliorated cognitive decline in the presence of FDG-PET hypometabolism. The cognitive reserve hypothesis is based on the observation that the actual cognitive performance is often not concordant with the existing neuronal injury. A substantial number of subjects show relatively stable cognitive performance despite advanced pathological brain alterations (Stern, 2009, Landau et al., 2011, Soldan et al., 2017), indicating that subjects with higher education can have lower FDG-PET metabolism to sustain their cognitive ability. In patients with FTD, few imaging studies showed a negative correlation of higher education with FDG-PET glucose metabolism, brain perfusion, or resting state fMRI assessed functional connectivity, controlling for cognitive performance (Perneczky et al., 2007, Perneczky et al., 2007, Borroni et al., 2009, Premi et al.,). Additionally, at the behavioural level, higher education attenuated the effect of hypoperfusion on the disinhibited phenotype in the bvFTD phenotype (Premi et al., 2013b). However, there is a dearth of data in FTD on the question whether higher education is associated with higher cognitive performance than expected based on the level of brain alterations in FTD, i.e. the core concept of reserve.

In order to address this question, we first established in patients with FTD the spatial pattern of FDG-PET hypometabolism that is associated with changes in global cognition. Consistent with previous studies (Ishii et al., 1998, Grimmer et al., 2004), we found decreased FDG-PET metabolism in frontal and temporal regions compared to healthy controls for the whole FTD cohort. Divided by the clinical phenotypes of FTD, there was a significant hypometabolism for both PPA and bvFTD when compared to HC. This was also reflected at the single patient level, since the majority of patients from both phenotype groups indicated FDG-PET results ranging below two standard deviations from the mean of healthy controls. There was no significant difference of FDG-PET quantification between PPA and bvFTD, highlighting the overlap of FDG-PET hypometabolism patterns between clinical phenotypes.

Only a subset of frontotemporal regions in the left lateral temporal cortex was associated with MMSE. We chose that cluster rather than all regions of FDG-PET metabolism in order to enhance the sensitivity of our analysis of reserve, i.e. only those brain alterations that are linked to global cognitive performance for our residualization approach. Quantitative FDG-PET metabolism in the left temporal cluster was significantly lower in both studied phenotypes of FTD when compared to HC, which again emphasizes the metabolic overlap of PPA and bvFTD.

We found that higher education is associated with higher MMSE score than expected based on the level of left temporal FDG-PET hypometabolism, suggesting that education is associated with a systematic deviation of global cognition from the level of cognitive performance that would be expected based on the severity of metabolic alterations. In other words, higher education was associated with higher resilience against the impact of regional hypometabolism on global cognition. We adopted the residualization approach which was first proposed as a quantitative index in patients with Alzheimer’s disease (Reed et al., 2010). This approach has been found to be useful as an index of reserve across several studies (Zahodne et al., 2013, Serra et al., 2017). One disadvantage of this approach is that the residual likely results from a mixture of random prediction error (e.g. due to measurement error) and a meaningful portion of variable that can be attributed to reserve. Despite this limitation, we found that education was associated with a positive deviation of MMSE residuals, supporting that protective factors may explain part of the portion of unexpectedly high cognitive performance despite manifest pathological brain alterations. Our findings are further supported by our auxiliary analyses, where education was associated with more severe FDG-PET metabolism when controlling for cognitive performance, suggesting that patients with higher education can tolerate more FDG-PET hypometabolism before showing a similar level of cognitive impairment as those patients with lower levels of education (Stern et al., 1992, STERN, 2002). When analysing the clinical phenotypes bvFTD and PPA separately, we found that residualized memory function was significantly correlated with the education level in patients with PPA patients but not in patients with bvFTD. The even stronger results in the separate analysis of patients with PPA excluded that the main findings of our study were driven by the mixture of phenotypes. The phenotype differences could be related to the smaller sample size of the bvFTD or to differences of the phenotype itself. In this regard, an autopsy study in FTD reported an opposite positive correlation of cognitive reserve (expressed by the education level and the occupation index) with higher grey matter density in several frontal regions and no correlation of higher grey matter density in any brain region with lower cognitive reserve (Placek et al., 2016). The most relevant difference between both studies was the predominant bvFTD phenotype in the autopsy study in contrast to the predominant PPA phenotype in the present work. In our cohort, we only found significant correlations with temporal regions and neither positive nor negative correlations with frontal regions. Thus, in summary there could be a variable impact of reserve indices on neuropathology in different brain networks (i.e. temporal vs. frontal) underlying the different FTD phenotypes.

The understanding of the systematic deviation of cognitive abilities from what would be expected based on manifest FDG-PET hypometabolism has clinical implications: when FDG-PET is judged normal or abnormal in the workup of suspected FTD in patients, where seemingly subtle FDG-PET alterations can be accompanied by relatively strong cognitive impairment, i.e. when subjects show low education. Low to moderate reduced left temporal glucose metabolism combined with a low education results in a higher likelihood of FTD compared to a patient with a high education level and the same cognitive performance.

This is especially relevant since subjects with FTD often present with mild but not severe cognitive impairment (also reflected by a mean MMSE of 24.0 ± 5.5 in the current cohort). In our sub-analysis investigating patients with lower/ higher education levels separately, we found both groups to show significant hypometabolism in frontal and temporal cortices when compared to healthy controls. This is in line with a former study in AD patients that compared the diagnostic accuracy of FDG-PET between groups with higher and lower education levels and found education not to be a major confounder (Mainta et al., 2018). Therefore, different education levels need to be considered when interpreting FDG-PET images in FTD patients, but the sensitivity of the method should be high enough to detect subjects with low education levels.

The paradigm of cognitive reserve is also of interest in terms of further cognitive deterioration. It has been hypothesized that cognitive reserve helps to longer maintain cognitive functions, but with faster decline over time (Stern, 2009). This hypothesis was confirmed in patients with AD, where higher cognitive reserve expressed by the discrepancies between neuronal injury biomarkers and cognitive performance led to faster decline in clinical follow-up (Beyer et al., 2019). In a longitudinal evaluation of cognitive decline in bvFTD patients, higher lifetime cognitive experience demonstrated more rapid decline on measures of executive function (Massimo et al., 2019), but an overall longer survival (Massimo et al., 2015). Further longitudinal measurements of the relationship between cognitive reserve and levels of hypometabolism will be of great interest in the field of FTD.

## 5 Limitations

As limitations of this study, we note that the years of education is not the only proxy that has been shown to assess cognitive reserve. Other individual factors such as life activities and occupation levels have been shown to contribute to the cognitive reserve but those were not recorded due to the retrospective design of the study (Stern et al., 1992, Maiovis et al., 2018, Alexander et al., 1997). Furthermore, data about family history which could indicate a genetic background and precise recordings of the disease duration were not available.

Furthermore, MMSE as the commonly used instrument for measuring cognitive impairment cannot replace detailed neuropsychological testing and does not represent all aspects of cognitive decline. Especially frontal lobe functions are poorly represented by the MMSE score which may explain the missing voxel-wise associations between cognitive function (expressed by MMSE) and left temporal hypometabolism in the bvFTD subgroup. Nevertheless, both clinical phenotypes overlap and bvFTD patients also showed a significantly lower left-temporal metabolism compared to HC. Therefore, this temporal involvement can also occur in bvFTD and might be better represented by the MMSE score. More detailed cognitive testing would be favorable, however due to lacking standardization of cognitive testing in our cross-sectional FTD cohort, we focused on commonly used MMSE to gain results relevant for the majority of clinical institutions. We note that although we included patients at all levels of cognitive impairment there could be a selection bias since FTD patients with severe cognitive impairment likely had not undergone FDG-PET imaging at their clinical workup.

## 6 Conclusions

In the hitherto largest mixed collective of clinical assessed FTD patients, the education level as a surrogate of cognitive performance explains discrepancies between left temporal FDG-PET hypometabolism and global cognition supporting the concept of an existing cognitive reserve. As already proposed for other neurodegenerative diseases, higher education levels could potentially modify cognitive deterioration also in FTD.

## CRediT authorship contribution statement

**Leonie Beyer:** Writing - original draft, Methodology, Formal analysis. **Johanna Meyer-Wilmes:** Methodology, Formal analysis. **Sonja Schönecker:** Validation. **Jonas Schnabel:** Validation. **Julia Sauerbeck:** Validation. **Maximilian Scheifele:** Validation. **Catharina Prix:** Validation. **Marcus Unterrainer:** Validation. **Cihan Catak:** Validation. **Oliver Pogarell:** Validation. **Carla Palleis:** Validation. **Robert Perneczky:** Validation. **Adrian Danek:** Validation. **Katharina Buerger:** Validation. **Peter Bartenstein:** . **Johannes Levin:** Validation. **Axel Rominger:** Validation. **Michael Ewers:** Conceptualization, Supervision. **Matthias Brendel:** Conceptualization, Writing - review & editing, Supervision, Methodology.
